# Seroprevalence of Measles and Mumps Antibodies Among Individuals With Cancer

**DOI:** 10.1001/jamanetworkopen.2021.18508

**Published:** 2021-07-28

**Authors:** Sara R. Marquis, Jennifer K. Logue, Helen Y. Chu, Tillie Loeffelholz, Z. Z. Quinn, Catherine Liu, F. Marc Stewart, Paul A. Carpenter, Steven A. Pergam, Elizabeth M. Krantz

**Affiliations:** 1 Vaccine and Infectious Disease Division, Fred Hutchinson Cancer Research Center, Seattle, Washington; 2 Division of Allergy and Infectious Diseases, University of Washington, Seattle; 3 Antimicrobial Stewardship, Seattle Cancer Care Alliance, Seattle, Washington; 4 Clinical Research Division, Fred Hutchinson Cancer Research Center, Seattle, Washington; 5 Department of Hematology and Hematopoietic Cell Transplantation, City of Hope, Duarte, California; 6 Bone Marrow Transplantation Outpatient Services, Seattle Cancer Care Alliance, Seattle, Washington; 7 Department of Pediatrics, University of Washington, Seattle; 8 Infection Prevention, Seattle Cancer Care Alliance, Seattle, Washington

## Abstract

**Importance:**

Although patients with cancer are at an increased risk of infection-related complications, few studies have characterized their vulnerability to measles and mumps. Given the recent outbreaks and increased community vaccine hesitancy, understanding measles and mumps immunity within this population is vital.

**Objectives:**

To identify a point prevalence estimate of protective measles and mumps antibodies among ambulatory patients with cancer.

**Design, Setting, and Participants:**

In this cross-sectional study, residual clinical plasma samples were obtained from consecutive patients with cancer at Seattle Cancer Care Alliance/Fred Hutchinson Cancer Research Center in Seattle, Washington, in August 2019. These samples were tested for measles and mumps IgG using a commercial enzyme-linked immunosorbent assay. Patients without cancer were excluded from the analysis.

**Exposures:**

Patient age, sex, self-reported race and ethnicity, primary disease, receipt of chemotherapy in the past 30 days before sample collection, hematopoietic cell transplant (HCT) history, and date of most recent intravenous immunoglobulin treatment were abstracted from electronic medical records.

**Main Outcomes and Measures:**

Measles and mumps IgG seroprevalence, defined as the proportion of patients with positive antibody test results, was measured overall and among the subgroups.

**Results:**

Of the 959 patients included in the analysis, 510 (53%) were male individuals and the mean (SD) age at sample collection was 60 (15) years. Most patients (576 [60%]) had a malignant solid tumor, and 383 patients (40%) had a hematologic malignant neoplasm; 146 patients (15%) had an HCT history. Overall, the seroprevalence of measles antibodies was 0.75 (95% CI, 0.72-0.78), and the seroprevalence of mumps antibodies was 0.62 (95% CI, 0.59-0.65). The lowest seroprevalences were among patients with a hematologic malignant neoplasm (0.63 for measles and 0.48 for mumps), those with a history of HCT (0.46 for measles and 0.29 for mumps), and those aged 30 to 59 years (0.49-0.63 for measles and 0.41-0.58 for mumps).

**Conclusions and Relevance:**

In this study, 25% of ambulatory patients with cancer lacked protective antibodies for measles and 38% lacked protective antibodies for mumps. Deficits in protective antibodies underscore patients’ increased risk during outbreaks and emphasize the need for community-based efforts to increase herd immunity to protect this population.

## Key Points

**Question:**

What is the seroprevalence of measles and mumps among patients with cancer?

**Findings:**

In this cross-sectional seroprevalence study that involved 959 patients with cancer, 25% of patients lacked protective antibodies for measles and 38% lacked antibodies for mumps. Younger patients (aged 30-59 years), those with hematologic malignant neoplasms, and recipients of a hematopoietic stem cell transplant had a significantly lower seroprevalence for both measles and mumps.

**Meaning:**

Low seroprevalence of measles and mumps among patients with cancer places them at increased risk for infection during measles or mumps outbreaks in the community.

## Teaser

This cross-sectional study examines serological test results for protective antibodies for measles and mumps among patients with hematologic malignant neoplasm or solid tumors.

## Introduction

Measles and mumps are highly contagious viral infections that were nearly eliminated after the introduction of the measles-mumps-rubella (MMR) vaccine in 1963.^1^ Recent decreases in vaccination rates, which are associated with vaccine hesitancy, have led to outbreaks across the United States.^2^

Individuals who are undergoing cancer treatment are at an increased risk for measles or mumps infection and life-threatening complications, such as viral pneumonia and encephalitis.^3^ A decrease in measles and mumps immunity has been reported among recipients of hematopoietic cell transplant (HCT) and children who underwent chemotherapy for acute lymphoblastic leukemia.^4,5^ Current guidelines recommend postponing vaccination for up to 2 years after undergoing an HCT or other immunosuppressive therapies, but humoral immunity can wane even after revaccination.^3,5^ Measles outbreaks in oncology wards are rare but have been associated with case fatality rates that are as high as 70%.^6^ Little is known about mumps among patients with cancer, especially in adult populations.

Despite immune deficits and increased infection-related mortality, seroprevalence of measles and mumps has not been well characterized among patients with cancer. Work by Guzek et al^7^ has suggested a lower seroprevalence of measles and mumps in patients with cancer compared with healthy control individuals, but additional seroprevalence estimates during the modern era of cancer treatment are needed. With the reemergence of these viruses and increased use of highly immunosuppressive therapies and biologics, there is a need to understand the seroprevalence among these populations, for whom the recommended herd immunity levels of 93% to 95% for measles and 88% to 92% for mumps may not be feasible.^8,9,10^ Such knowledge may aid cancer centers in developing better prevention and response strategies for outbreaks, which have been increasingly reported worldwide. These data may also inform screening programs that target patient populations with lower seroprevalence for revaccination.^11^

In this cross-sectional study, we performed serological tests to identify a point prevalence estimate of protective measles and mumps antibodies among a large cohort of ambulatory patients with cancer, and we compared the seroprevalence among demographic and clinical subgroups. We hypothesized that patients with cancer would have a lower seroprevalence of measles and mumps antibodies compared with the general population and would therefore increase the risk of cancer centers for outbreaks of these vaccine-preventable diseases.

## Methods

This cross-sectional study was approved by the Fred Hutchinson Cancer Research Center Institutional Review Board. We obtained a waiver of informed consent because the study met the US Food and Drug Administration criteria. Access to plasma samples and electronic medical records was obtained under the waiver of consent approved by the institutional review board. We followed the Strengthening the Reporting of Observational Studies in Epidemiology (STROBE) reporting guideline.

### Sample and Data Collection

The study population consisted of consecutive outpatients who received care at the Seattle Cancer Care Alliance/Fred Hutchinson Cancer Research Center and had residual plasma samples available after routine clinical testing over a 5-day period in August 2019. The Seattle Cancer Care Alliance/Fred Hutchinson Cancer Research Center is a large ambulatory cancer center in Seattle, Washington, that serves patients from multiple states in the Pacific Northwest region as well as patients from across the United States who were referred to the center for HCT, immunotherapy, and other research protocols. The facility provides services for more than 75 000 outpatient visits yearly.

Patient age, sex, self-reported race and ethnicity, primary disease, date of most recent intravenous immunoglobulin (IVIG) treatment, receipt of chemotherapy in the past 30 days before sample collection, and HCT history were abstracted from center-based electronic medical records. Patients without cancer were excluded from analysis.

### Laboratory Testing

Specimens were collected and separated into 3 aliquots and stored at −80 °C at the University of Washington in Seattle. Enzyme-linked immunosorbent assay kits (Genway Biotech Inc) were used to detect and quantify measles and mumps IgG antibodies from plasma. Plasma samples were run in duplicate. Absorbance was read at 450 nm against the reagent blank using a microplate spectrophotometer (Epoch Microplate Spectrophotometer; BioTek Instruments, Inc). The IgG antibody concentration (units per milliliter) was calculated using a standard curve, generated by graphing absorptions of the standards drawn point to point against their concentrations. Plasma samples were categorized as positive (≥12 U/mL), equivocal (>8 to <12 U/mL), or negative (≤8 U/mL) in accordance with the manufacturer’s fixed cutoff standard.

### Statistical Analysis

Seroprevalence for measles and mumps was defined as the proportion of patients with positive antibody test results; equivocal antibody test results were not considered protective. Overall and subgroup seroprevalences were estimated with Wilson 95% CIs. Poisson multivariable regression with robust SEs was used to compare subgroups and estimate prevalence ratios (PRs). Models were adjusted for age group, sex, primary disease (hematologic malignant neoplasm vs solid tumor), HCT history (none, ≤1 year, or >1 year before sample collection), chemotherapy in the 30 days before sample collection, and receipt of IVIG (none, ≤16 weeks, or >16 weeks before sample collection). Categories for HCT history were chosen to represent early- vs mid- and late-stage survivors of HCT, and categories for the most recent IVIG treatment were based on a 16-week time point that represented 4 or more half-lives for circulating IgG.^12^

We conducted 2 sensitivity analyses to address the association between age and disease type. In the first sensitivity analysis, we examined regression models that included an interaction between age and disease type to assess whether the association between age and seroprevalence varied by disease type and whether the association between disease type and seroprevalence varied by age group. In the second sensitivity analysis, because of concerns that the association between younger age and seroprevalence may be mediated or explained by underlying disease, we examined multivariable models in which primary disease and HCT history were excluded. We also conducted sensitivity analyses in which equivocal test results were categorized as positive test results for seroprevalence estimates. Missing data were categorized as unknown for descriptive tables. SAS, version 9.4 (SAS Institute), was used for analyses.

## Results

Plasma samples were collected from 1001 unique patients; 1 patient was excluded because of a transcription error, and 41 patients did not have cancer. The Table shows the demographic and clinical characteristics of the 959 eligible patients. The mean (SD) age at sample collection was 60 (15) years; 5 patients were younger than 18 years. The study included 510 male (53%) and 449 female (47%) patients. A wide variety of cancer diagnoses were represented, but most patients (576 [60%]) had a malignant solid tumor, with breast (127 [13%]) and gastrointestinal (156 [16%]) cancers being the most common. Hematologic malignant neoplasms were present in 383 patients (40%). A small number of patients (146 [15%]) had a history of HCT, and 310 (32%) had received chemotherapy in the 30 days before sample collection.

**Table.** Baseline Demographic and Clinical Characteristics of Study Cohort^a^

Characteristic	No. (%)
No. of patients	959
Age at sample collection, mean (SD), y	60 (15)
Age group, y	
<30	38 (4)
30-39	65 (7)
40-49	103 (11)
50-59	222 (23)
60-69	279 (29)
70-79	193 (20)
≥80	59 (6)
Sex	
Male	510 (53)
Female	449 (47)
Race	
White	780 (81)
Black or African American	37 (4)
Asian	90 (9)
Native Hawaiian or other Pacific Islander	7 (1)
American Indian or Alaska Native	13 (1)
Unknown	32 (3)
Ethnicity	
Not Hispanic or Latino	870 (91)
Hispanic or Latino	45 (5)
Unknown	44 (5)
Primary cancer	
Brain or spinal	2 (<1)
Breast	127 (13)
Endocrine	14 (1)
Gastrointestinal	157 (16)
Genitourinary and renal	109 (11)
Gynecological	7 (1)
Lung, thoracic, and head and neck	62 (6)
Sarcoma	41 (4)
Skin	57 (6)
Hematologic malignant neoplasm	383 (40)
Primary disease	
Solid tumor	576 (60)
Hematologic malignant neoplasm	383 (40)
HCT history, before sample collection	
None	813 (85)
≤1 y	76 (8)
>1 y to 2 y	22 (2)
>2 y	48 (5)
Time from most recent HCT to sample collection, median (range), d	342 (2-9043)
Date of most recent IVIG treatment, before sample collection	
None	921 (96)
0-8 wk	5 (1)
>8-16 wk	3 (<1)
>16 wk	30 (3)
Receipt of chemotherapy in past 30 d before sample collection	
Any oral or IV chemotherapy^b^	310 (32)
Any oral chemotherapy^b^	247 (26)
Any IV chemotherapy	87 (9)
Time from most recent IV chemotherapy to sample collection, median (range), d^c^	7 (0-30)
No. of days with IV chemotherapy in 30 d before sample collection, median (range)^c^	2 (1-16)

Abbreviations: HCT, hematopoietic cell transplant; IV, intravenous; IVIG, intravenous immunoglobulin.

^a^ Patients without cancer who were excluded from the original cohort had the following primary diseases or conditions: amyloidosis, aplastic anemia, idiopathic thrombocytopenia, sickle cell anemia, common variable immunodeficiency, HCT donor, Gaucher disease, Rosai-Dorfman disease, mastocytosis, Diamond-Blackfan anemia, chronic granulomatous disease, Fanconi anemia, hemochromatosis, idiopathic CD4 lymphocytopenia, iron overload, paroxysmal nocturnal hemoglobinuria, protein C and S deficiency, SDHB gene mutation, severe combined immunodeficiency, thrombosis, thrombotic thrombocytopenic purpura, Wiskott-Aldrich syndrome.

^b^ Oral chemotherapy data were based on prescription ordered; thus, use was presumed only.

^c^ Among those who received IV chemotherapy in 30 days before sample collection.

### Seroprevalence of Measles Antibodies

**Figure 1.**
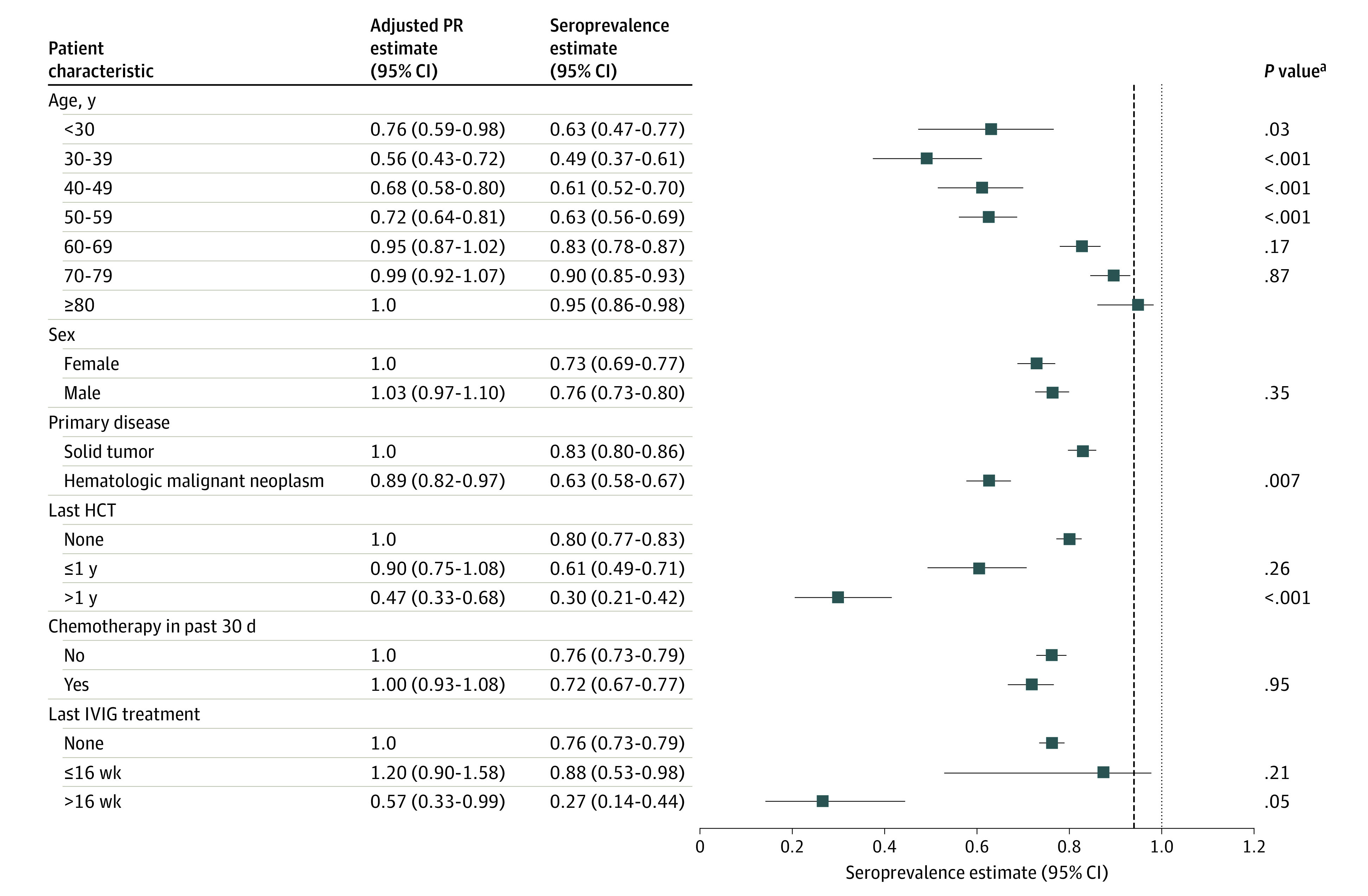
Measles Seroprevalence and Adjusted Prevalence Ratio (PR) Estimates by Subgroup Squares represent measles seroprevalence estimates, and the error bars show the 95% CIs for these estimates. The vertical dashed line shows the middle value (0.94) for the recommended range required for herd immunity (0.93-0.95). The PR estimates from a multivariable model were adjusted for age group, sex, primary disease, hematopoietic cell transplant (HCT) history before sample collection, chemotherapy in the 30 days before sample collection, and intravenous immunoglobulin (IVIG) treatment before sample collection. ^a^The *P* values correspond to the adjusted PR estimates.

**Figure 2.**
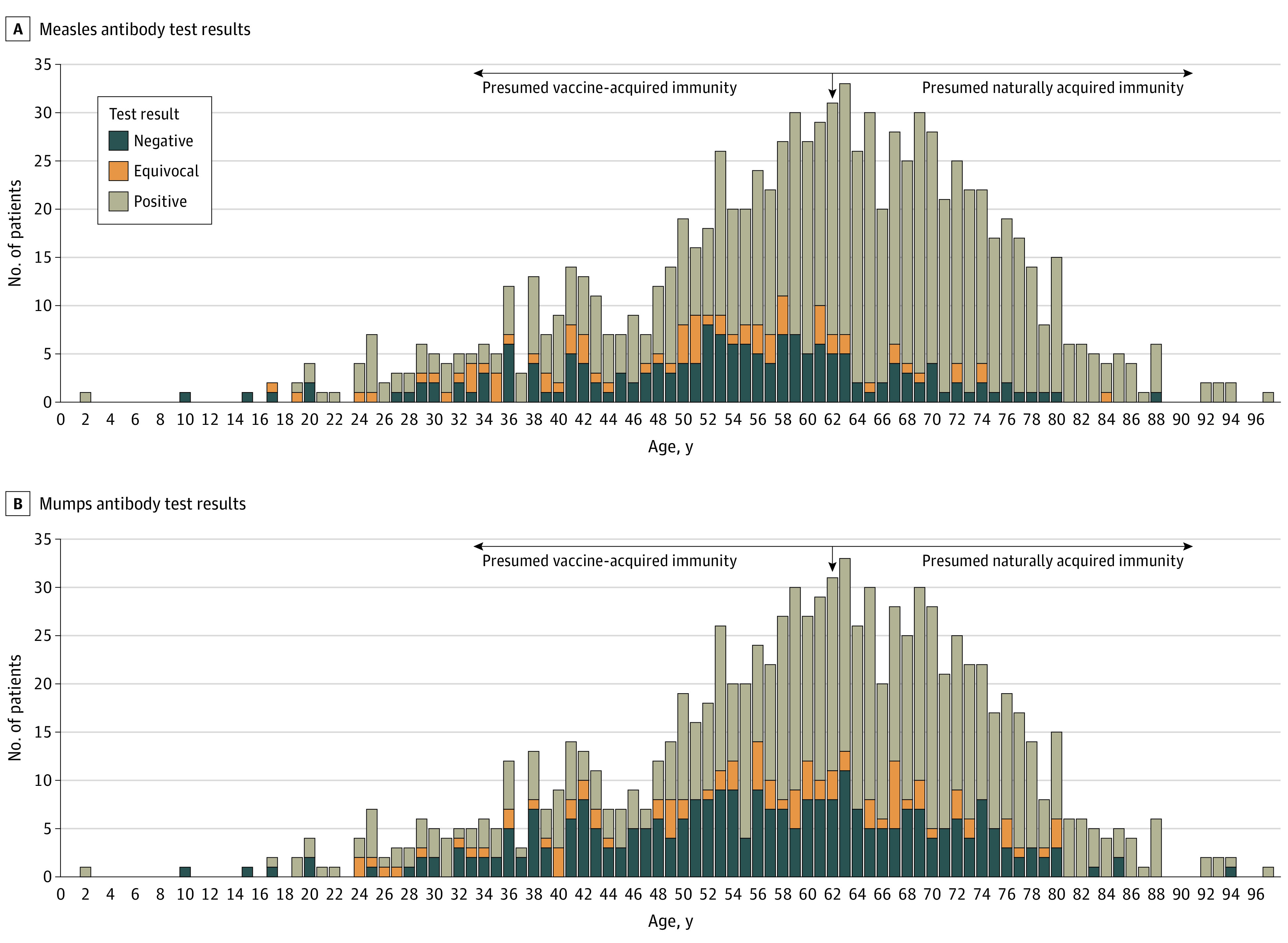
Distribution of Age at Sample Collection and Measles and Mumps IgG Antibody Test Results Total height of filled bars indicates the frequency of patient age in the study cohort, with the height of the blue, orange, and gray bars representing the number of patients with negative, equivocal, or positive test results. The vertical arrowhead points to those born in 1957 (age 62 years at sample collection). Those older than 62 years (under right arrow) were older than 5 years at the introduction of the measles-mumps-rubella vaccine and alive when measles and mumps were common and were therefore presumed to have naturally acquired immunity. Those younger than 62 years (under left arrow) were presumed to have vaccine-acquired immunity.^13,14^

Of the 959 patients analyzed, 718 (75%) had a positive measles antibody test result, 74 (8%) had an equivocal test result, and 167 (17%) had a negative test result. Overall, the seroprevalence of measles antibodies was 0.75 (95% CI, 0.72-0.78). Estimates among the demographic and clinical subgroups are shown in Figure 1. Seroprevalence varied among age groups, with estimates as low as 0.49 (95% CI, 0.37-0.61) among those aged 30 to 39 years and as high as 0.95 (95% CI, 0.86-0.98) among those 80 years or older. We observed a higher level of seropositivity in patients who were born before 1957 (62 years or older at the time of sample collection). This population, in general, was presumed to have naturally acquired immunity by the Centers for Disease Control and Prevention because individuals in the group were older than 5 years at the 1963 introduction of the MMR vaccine and were alive during an era when measles and mumps infection were common (Figure 2).^13,14^ The estimate for measles seroprevalence was lower among patients with hematologic malignant neoplasms (0.63; 95% CI, 0.58-0.67) than among patients with solid tumors (0.83; 95% CI, 0.80-0.86). Patients with a history of HCT also exhibited lower seroprevalence (0.46; 95% CI, 0.38-0.54).

Prevalence ratio estimates for measles seroprevalence from multivariable models adjusted for age group, sex, primary disease, HCT history, receipt of chemotherapy in the past 30 days, and date of most recent IVIG treatment before sample collection are shown in Figure 1. Many of the univariable subgroup patterns described earlier persisted in the multivariable models. Compared with those 80 years or older, patients who were younger than 30 years (PR, 0.76; 95% CI, 0.59-0.98), those aged 30 to 39 years (PR, 0.56; 95% CI, 0.43-0.72), those aged 40 to 49 years (PR, 0.68; 95% CI, 0.58-0.80), and those aged 50 to 59 years (PR, 0.72; 95% CI, 0.64-0.81) had significantly lower seroprevalence. Patients with hematologic malignant neoplasms had a significantly lower seroprevalence compared with those with solid tumors (PR, 0.89; 95% CI, 0.82-0.97). An HCT history of 1 year or more before sample collection was associated with 50% lower seroprevalence compared with no previous HCT (PR, 0.47; 95% CI, 0.33-0.68). Prevalence of measles antibodies did not vary significantly by sex or chemotherapy in the previous 30 days before sample collection.

### Seroprevalence of Mumps Antibodies

**Figure 3.**
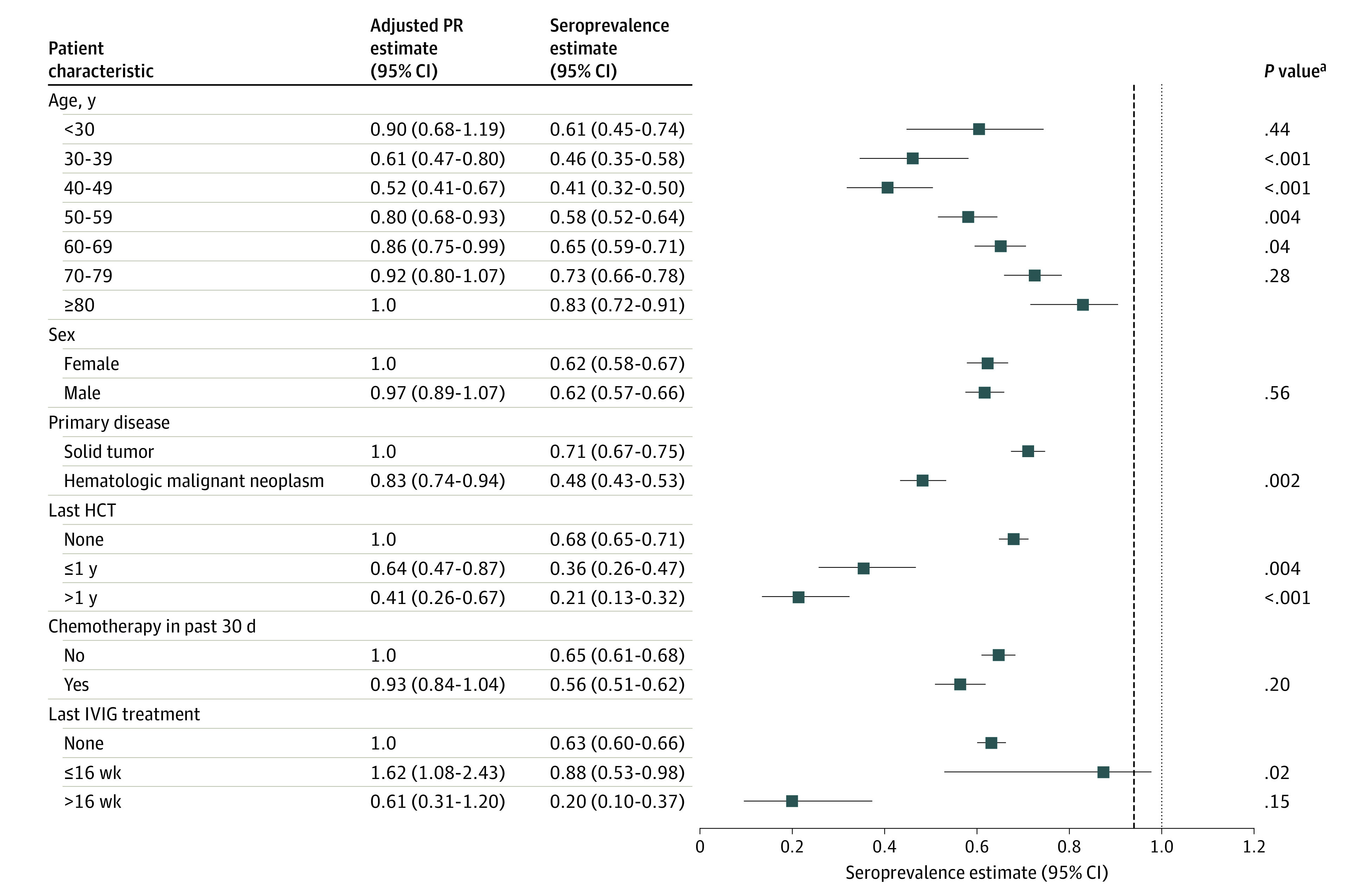
Mumps Seroprevalence and Adjusted Prevalence Ratio (PR) Estimates by Subgroup Squares represent mumps seroprevalence estimates, and error bars show the 95% CIs for these estimates. The vertical dashed line shows the middle value (0.90) for the recommended range required for herd immunity (0.88-0.92). The PR estimates from a multivariable model were adjusted for age group, sex, primary disease, hematopoietic cell transplant (HCT) history before sample collection, chemotherapy in the 30 days before sample collection, and intravenous immunoglobulin (IVIG) treatment before sample collection. ^a^The *P* values correspond to the adjusted PR estimates.

A total of 595 patients (62%) had a positive mumps antibody test result, 90 (9%) had an equivocal test result, and 274 (29%) had a negative test result. Overall, the seroprevalence of mumps antibodies was 0.62 (95% CI, 0.59-0.65). Similar to measles seropositivity, mumps seropositivity was increased in those older than 62 years, who were also presumed to have naturally acquired immunity (Figure 2).^13,14^ We observed patterns in the subgroup estimates for mumps that were similar to those for measles, although the seroprevalence estimates were consistently lower (Figure 3). Similar to measles, the lowest seroprevalences were among patients with hematologic malignant neoplasms (0.48; 95% CI 0.43-0.53), those with a history of HCT (0.29; 95% CI 0.22, 0.37), and those aged 30-59 years (0.41-0.58) (Figure 3).

Multivariable models showed associations similar to those observed for measles seroprevalence (Figure 3). Patients aged 30 to 59 years had significantly lower seroprevalence than patients 80 years or older. In contrast to measles seroprevalence, the youngest age group (<30 years) did not have significantly lower mumps seroprevalence (PR, 0.90; 95% CI, 0.68-1.19), whereas patients aged 60 to 69 years had significantly lower mumps seroprevalence (PR, 0.86; 95% CI, 0.75-0.99) compared with the oldest age group (≥80 years). Patients with hematologic malignant neoplasms had a significantly lower seroprevalence than those with solid tumors (PR, 0.83; 95% CI, 0.74-0.94). An HCT history was also associated with lower seroprevalence (≤1 year with HCT vs no HCT: PR, 0.64 [95% CI, 0.47-0.87]; >1 year with HCT vs no HCT: PR, 0.41 [95% CI, 0.26-0.67]).

### Sensitivity Analyses

In the first sensitivity analysis, for measles, multivariable models showed a significant interaction between age group and disease type, suggesting that the differences in seroprevalence between younger patients and those 80 years or older were generally more pronounced among patients with hematologic malignant neoplasms than patients with solid tumors. Specifically, the PR for patients aged 40 to 49 years vs 80 years or older was 0.42 (95% CI 0.27-0.64) among patients with hematologic malignant neoplasms and 0.80 (95% CI 0.67-0.94) among those with solid tumors; the PR for patients aged 60 to 69 years vs 80 years or older was 0.83 (95% CI 0.73-0.94) among patients with hematologic malignant neoplasms and 1.0 (95% CI, 0.90-1.10) among those with solid tumors; the PR for patients aged 70 to 79 years vs 80 years or older was 0.89 (95% CI, 0.78-1.01) among patients with hematologic malignant neoplasms and 1.04 (95% CI, 0.95-1.14) among those with solid tumors (eFigure 1 in the Supplement). In addition, the lower seroprevalence among patients with hematologic malignant neoplasms compared with those with solid tumors was mainly observed among patients aged 40 to 49 years (PR, 0.56; 95% CI, 0.36-0.88). For mumps, the interaction between age group and disease type was not significant. Estimates from that model are shown in eFigure 2 in the Supplement.

In the second sensitivity analysis, disease type and HCT history were excluded from the models because of concerns that these variables may be a mechanism that could explain an association between young age and seroprevalence (eTables 1 and 2 in the Supplement). In these models, estimates for the youngest age group were similar to estimates in the main analysis for measles but differed for mumps, with patients younger than 30 years having significantly lower seroprevalence compared with those 80 years or older in the sensitivity model only (PR, 0.73 [95% CI, 0.55-0.96] in the second sensitivity model vs PR, 0.90 [95% CI, 0.68-1.19] in the main analysis).

We also computed estimates of seroprevalence by counting equivocal test results as positive instead of negative test results (eTable 3 in the Supplement). Although estimates were slightly higher in this case (measles seroprevalence, 0.83 [95% CI, 0.80-0.85]; mumps seroprevalence, 0.71 [95% CI, 0.68-0.74]), they remained at suboptimal levels for protection.

## Discussion

To our knowledge, this study is one of the first studies to measure measles and mumps seroprevalence among patients with cancer in the modern era of cancer treatment. We found that a quarter of patients with cancer in this study (8% with equivocal and 17% with negative test results) lacked protective measles antibodies and more than one-third (9% with equivocal and 29% with negative test results) lacked mumps antibodies protection. These data suggest an increased risk for this patient population compared with the general population, in which approximately 8% lack protective antibodies for measles and 13% for mumps.^1^ Furthermore, we identified subgroups in whom deficits in immunity were most common: patients aged 30 to 59 years, patients with hematologic malignant neoplasms, and patients who had received an HCT.

Hematologic malignant neoplasms lead to known immune deficits; therefore, low seroprevalence in this subgroup was not unexpected. In Europe, Guzek et al^7^ found a similar lower seroprevalence for both measles and mumps among those with hematologic malignant neoplasms compared with either patients with solid malignant neoplasms or healthy control individuals. We observed lower seroprevalence among recipients of an HCT, especially those who underwent the HCT more than 1 year before the sample collection, a finding that is similar to reports from previous research.^5^

We observed heterogeneity in seroprevalence across the age groups in this cohort. Patients between 30 and 59 years of age showed significantly lower seroprevalence for both measles and mumps compared with those 80 years or older when adjusting for other variables, including type of malignant neoplasm and HCT history. Children and young adults (<30 years) had significantly lower seroprevalence for measles but not mumps compared with adults 80 years or older in fully adjusted models. However, sensitivity models that did not adjust for primary disease type and HCT history demonstrated lower mumps seroprevalence in children and young adults compared with adults 80 years or older. This finding suggests that the association of young age with mumps seroprevalence may be explained by the predisposition of younger patients with cancer to have a disease or treatment (such as HCT) associated with a lower level of protective antibodies. Thus, this younger age group still warrants important consideration for deficits in both measles and mumps immunity. The higher seropositivity estimates for age groups corresponded to estimates for those who were presumed, according to the Centers for Disease Control and Prevention vaccination guidelines, to have naturally acquired disease (born before 1957) vs those who were more likely to have vaccine-acquired immunity (born after 1957).^13^ For measles, this finding aligns with recent research in healthy adult populations that similarly reported decreased measles seroprevalence in vaccinated vs naturally infected individuals.^15,16^

Studies in pediatric patients with cancer have shown the adverse outcome of chemotherapy for measles and mumps immunity.^17,18^ We did not see a similar consequence of recent chemotherapy for seroprevalence in this study’s predominantly adult population, suggesting potential differences in immune response, disease groups, and intensity or type of chemotherapy between adults and children.

Low seroprevalence among patients with cancer is concerning for many reasons. In the United States, the increase in vaccine hesitancy and weak regulations for vaccination of school-aged children place many communities below the protective thresholds and have been factors in recent outbreaks.^2^ With multiple community outbreaks in recent years, these data suggest that cancer centers are at particular risk.^19,20,21,22,23^ Hospitals and clinics with large numbers of patients with cancer could serve as epicenters of nosocomial transmission, or places in which a single case among a high-risk patient or staff member could lead to considerable morbidity and mortality.^6,24,25^ Furthermore, because immunosuppressed patients can present atypically (without rash or with isolated end-organ manifestations), delayed diagnoses can contribute to increased spread.^3^

The highly contagious nature of measles and mumps combined with the vulnerability of patients with cancer observed in this study highlight the need to increase community immunity in the United States. Efforts to increase vaccine education across diverse populations and to improve vaccine policy and support of childhood vaccination can help protect those who either have limited benefit from or cannot receive MMR vaccination. In addition, findings from this study underscore the need for national standards that require MMR vaccination or documented seropositivity among health care practitioners who work with patients with cancer.

### Limitations

This study has several limitations. First, antibody testing is often used as a surrogate for immunity, but IgG seropositivity may not adequately measure actual immunity, especially for mumps.^1^ Given the complex immune responses necessary for protection, true immunity levels may be underestimated. We were also unable to confirm MMR vaccination history among most patients and were therefore unable to draw conclusions regarding the association of vaccination (or revaccination) with seroprevalence in this cohort. However, this limitation is common in most seroprevalence studies of vaccine-preventable diseases, in which vaccine recall can be inaccurate even among pediatric populations.^26^ Second, the study population included only patients during a specific period who underwent clinical blood draws from which residual plasma samples were available for testing. Although we tested the samples from all consecutive patients available during this period at a laboratory that serves all clinics within the cancer center, we may not have fully captured every patient who did not undergo blood draws as part of their routine clinical care (ie, patients returning for cancer survivorship). Furthermore, this approach limited our ability to look at the differences between specific cancer subtypes and treatments. In addition, the cancer center cares for a small pediatric population (primarily for HCT), which hampered our ability to include these young patients.

## Conclusions

Protective measles antibodies were lacking in a quarter of patients with cancer in this study, and protective mumps antibodies were lacking for more than one-third of these patients. Such deficits in immunity were most common in those aged 30 to 59 years, who had hematologic malignant neoplasms and who underwent an HCT. These findings underscore these patients’ high risk during measles and mumps outbreaks and the need to increase herd immunity in the community.

## Notes

Supplement. **eTable 1.** Main Analysis and Sensitivity Analysis Multivariable Model Estimates for Associations with Measles Seroprevalence **eTable 2.** Main Analysis and Sensitivity Analysis Multivariable Model Estimates for Associations with Mumps Seroprevalence **eTable 3.** Measles and Mumps Seroprevalence Estimates by Subgroup, Counting Equivocal Results as Positive **eFigure 1.** Measles Seroprevalence Estimates and Adjusted Prevalence Ratio (PR) Estimates by Subgroup, for Sensitivity Analysis Including an Interaction Between Age Group and Disease Type **eFigure 2.** Mumps Seroprevalence Estimates and Adjusted Prevalence Ratio (PR) Estimates by Subgroup, for Sensitivity Analysis Including an Interaction Between Age Group and Disease Type
