# Supplementary material for: Seroprevalence of Measles and Mumps Antibodies Among Individuals With Cancer
Source: JAMA Netw Open. 2021 Jul 28;4(7):e2118508. doi: 10.1001/jamanetworkopen.2021.18508 (PMC8319758; doi:10.1001/jamanetworkopen.2021.18508)
Supplement: Supplement. — eTable 1. Main Analysis and Sensitivity Analysis Multivariable Model Estimates for Associations with Measles Seroprevalence eTable 2. Main Analysis and Sensitivity Analysis Multivariable Model Estimates for Associations with Mumps Seroprevalence eTable 3. Measles and Mumps Seroprevalence Estimates by Subgroup, Counting Equivocal Results as Positive eFigure 1. Measles Seroprevalence Estimates and Adjusted Prevalence Ratio (PR) Estimates by Subgroup, for Sensitivity Analysis Including an Interaction Between Age Group and Disease Type eFigure 2. Mumps Seroprevalence Estimates and Adjusted Prevalence Ratio (PR) Estimates by Subgroup, for Sensitivity Analysis Including an Interaction Between Age Group and Disease Type [file jamanetwopen-e2118508-s001.pdf]

Supplementary Online Content

Marquis SR, Logue JK, Chu HY, et al. Seroprevalence of measles and mumps antibodies among individuals with cancer. *JAMA Netw Open*. 2021;4(7):e2118508. doi:10.1001/jamanetworkopen.2021.18508

eTable 1. Main Analysis and Sensitivity Analysis Multivariable Model Estimates for Associations with Measles Seroprevalence

eTable 2. Main Analysis and Sensitivity Analysis Multivariable Model Estimates for Associations with Mumps Seroprevalence

eTable 3. Measles and Mumps Seroprevalence Estimates by Subgroup, Counting Equivocal Results as Positive

eFigure 1. Measles Seroprevalence Estimates and Adjusted Prevalence Ratio (PR) Estimates by Subgroup, for Sensitivity Analysis Including an Interaction Between Age Group and Disease Type

eFigure 2. Mumps Seroprevalence Estimates and Adjusted Prevalence Ratio (PR) Estimates by Subgroup, for Sensitivity Analysis Including an Interaction Between Age Group and Disease Type

This supplementary material has been provided by the authors to give readers additional information about their work.

© 2021 Marquis SR et al. *JAMA Network Open.*

eTable 1. Main Analysis and Sensitivity Analysis Multivariable Model Estimates for Associations with Measles Seroprevalence[a]

Variable	Main Analysis Model[b]		Sensitivity Analysis Model[c]	
	Adjusted PR (95% CI)	p-value	Adjusted PR (95% CI)	p-value
**Age, years**				
0-29	0.8 (0.6, 1.0)	0.03	0.7 (0.5, 0.9)	0.003
30-39	0.6 (0.4, 0.7)	<.001	0.5 (0.4, 0.7)	<.001
40-49	0.7 (0.6, 0.8)	<.001	0.7 (0.6, 0.8)	<.001
50-59	0.7 (0.6, 0.8)	<.001	0.7 (0.6, 0.8)	<.001
60-69	0.9 (0.9, 1.0)	0.17	0.9 (0.8, 1.0)	0.01
70-79	1.0 (0.9, 1.1)	0.87	1.0 (0.9, 1.0)	0.33
80+	1.0 (Reference)		1.0 (Reference)	
**Sex**				
Female	1.0 (Reference)		1.0 (Reference)	
Male	1.0 (1.0, 1.1)	0.35	1.0 (0.9, 1.1)	0.80
**Primary Disease**				
Solid tumor	1.0 (Reference)			
Hematological malignancy	0.9 (0.8, 1.0)	0.007		
**Hematopoietic Cell Transplant History**				
None	1.0 (Reference)			
HCT in past year	0.9 (0.7, 1.1)	0.26		
HCT >1 year ago	0.5 (0.3, 0.7)	<.001		
**Chemotherapy in past 30 days**				
No	1.0 (Reference)		1.0 (Reference)	
Yes	1.0 (0.9, 1.1)	0.95	1.0 (0.9, 1.1)	0.64
**Most Recent IVIG**				
None	1.0 (Reference)		1.0 (Reference)	
Within 16 weeks before sample	1.2 (0.9, 1.6)	0.21	1.2 (0.9, 1.5)	0.26
>16 weeks before sample	0.6 (0.3, 1.0)	0.045	0.4 (0.2, 0.6)	<.001

[a] Abbreviations, PR = prevalence ratio, CI = confidence interval, HCT = hematopoietic cell transplant, IVIG = intravenous immunoglobulin treatment.

[b] Adjusted for age group, sex, primary disease, transplant history, chemotherapy, and IVIG treatment.

[c] Adjusted for age group, sex, chemotherapy, and IVIG treatment.

© 2021 Marquis SR et al. *JAMA Network Open.*

eTable 2. Main Analysis and Sensitivity Analysis Multivariable Model Estimates for Associations with Mumps Seroprevalence[a]

Variable	Main Analysis Model[b]		Sensitivity Analysis Model[c]	
	Adjusted PR (95% CI)	p-value	Adjusted PR (95% CI)	p-value
**Age, years**				
0-29	0.9 (0.7, 1.2)	0.44	0.7 (0.6, 1.0)	0.03
30-39	0.6 (0.5, 0.8)	<.001	0.6 (0.4, 0.7)	<.001
40-49	0.5 (0.4, 0.7)	<.001	0.5 (0.4, 0.6)	<.001
50-59	0.8 (0.7, 0.9)	0.004	0.7 (0.6, 0.8)	<.001
60-69	0.9 (0.8, 1.0)	0.04	0.8 (0.7, 0.9)	0.002
70-79	0.9 (0.8, 1.1)	0.28	0.9 (0.8, 1.0)	0.10
80+	1.0 (Reference)		1.0 (Reference)	
**Sex**				
Female	1.0 (Reference)		1.0 (Reference)	
Male	1.0 (0.9, 1.1)	0.56	0.9 (0.9, 1.0)	0.19
**Primary Disease**				
Solid tumor	1.0 (Reference)			
Hematological malignancy	0.8 (0.7, 0.9)	0.002		
**Hematopoietic Cell Transplant History**				
None	1.0 (Reference)			
HCT in past year	0.6 (0.5, 0.9)	0.004		
HCT >1 year ago	0.4 (0.3, 0.7)	<.001		
**Chemotherapy in past 30 days**				
No	1.0 (Reference)		1.0 (Reference)	
Yes	0.9 (0.8, 1.0)	0.20	0.9 (0.8, 1.0)	0.06
**Most Recent IVIG**				
None	1.0 (Reference)		1.0 (Reference)	
Within 16 weeks before sample	1.6 (1.1, 2.4)	0.02	1.5 (1.0, 2.3)	0.03
>16 weeks before sample	0.6 (0.3, 1.2)	0.15	0.3 (0.2, 0.7)	0.002

[a] Abbreviations, PR = prevalence ratio, CI = confidence interval, HCT = hematopoietic cell transplant, IVIG = intravenous immunoglobulin treatment. Orange highlighted cells indicate the youngest age group estimates with both changes in statistical significance and changes of >20% in coefficient.

[b] Adjusted for age group, sex, primary disease, transplant history, chemotherapy, and IVIG treatment.

[c] Adjusted for age group, sex, chemotherapy, and IVIG treatment.

© 2021 Marquis SR et al. *JAMA Network Open.*

eTable 3. Measles and Mumps Seroprevalence Estimates by Subgroup, Counting Equivocal Results as Positive[a]

Subgroup	Measles Seroprevalence (95% CI)	Mumps Seroprevalence (95% CI)
**Age at sample collection, years**		
<30	0.76 (0.61 - 0.87)	0.76 (0.61 - 0.87)
30-39	0.71 (0.59 - 0.80)	0.57 (0.45 - 0.68)
40-49	0.73 (0.64 - 0.80)	0.56 (0.47 - 0.65)
50-59	0.74 (0.68 - 0.79)	0.68 (0.61 - 0.73)
60-69	0.87 (0.83 - 0.91)	0.75 (0.69 - 0.79)
70-79	0.92 (0.87 - 0.95)	0.78 (0.72 - 0.83)
80+	0.97 (0.88 - 0.99)	0.88 (0.77 - 0.94)
**Sex**		
Female	0.83 (0.79 - 0.86)	0.73 (0.69 - 0.77)
Male	0.82 (0.79 - 0.85)	0.70 (0.66 - 0.74)
**Primary disease**		
Solid Tumor	0.90 (0.87 - 0.92)	0.81 (0.78 - 0.84)
Hematologic Malignancy	0.72 (0.67 - 0.76)	0.57 (0.52 - 0.62)
**Recency of prior hematopoietic cell transplants**		
No prior transplant	0.87 (0.85 - 0.89)	0.77 (0.74 - 0.80)
Transplant ≤1 year before sample collection	0.79 (0.69 - 0.87)	0.49 (0.38 - 0.60)
Transplant >1 year before sample collection	0.34 (0.24 - 0.46)	0.27 (0.18 - 0.39)
**Prior IVIG**		
IVIG in 0-16 weeks before sample collection	1.00 (0.68 - 1.00)	0.88 (0.53 - 0.98)
IVIG in >16 weeks before sample collection	0.40 (0.25 - 0.58)	0.27 (0.14 - 0.44)
No IVIG before sample collection	0.84 (0.81 - 0.86)	0.73 (0.70 - 0.76)
**Oral or IV chemotherapy in 30 days before sample collection**		
No	0.83 (0.80 - 0.86)	0.73 (0.69 - 0.76)
Yes	0.81 (0.76 - 0.85)	0.68 (0.63 - 0.73)

[a]Seroprevalence defined by positive or equivocal result. Abbreviations: CI: confidence interval; IVIG: intravenous immunoglobulin treatment; IV: intravenous.

© 2021 Marquis SR et al. *JAMA Network Open.*

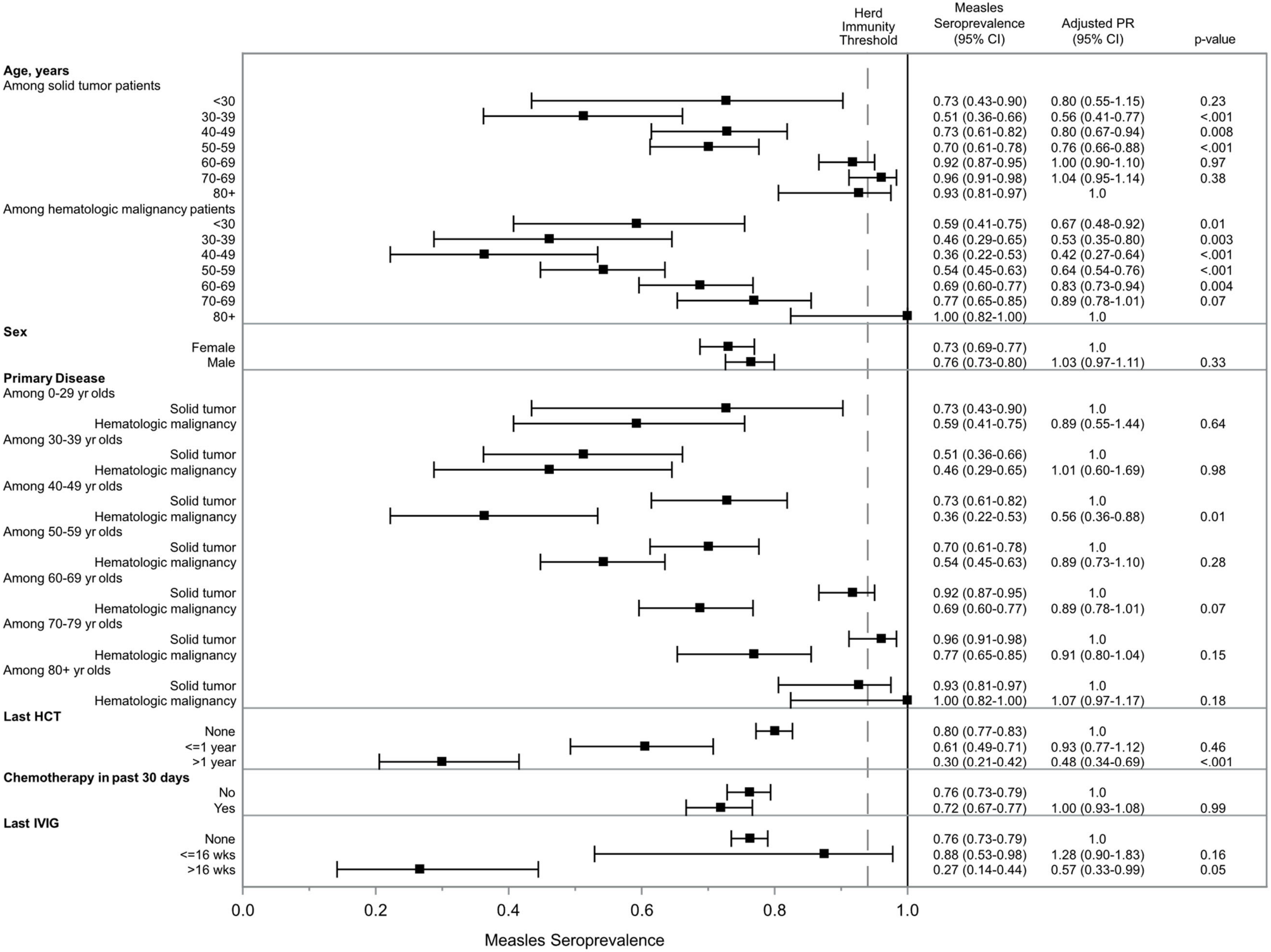

	Measles Seroprevalence (95% CI)	Adjusted PR (95% CI)	p-value
**Age, years**			
Among solid tumor patients			
<30	0.73 (0.43-0.90)	0.80 (0.55-1.15)	0.23
30-39	0.51 (0.36-0.66)	0.56 (0.41-0.77)	<.001
40-49	0.73 (0.61-0.82)	0.80 (0.67-0.94)	0.008
50-59	0.70 (0.61-0.78)	0.76 (0.66-0.88)	<.001
60-69	0.92 (0.87-0.95)	1.00 (0.90-1.10)	0.97
70-69	0.96 (0.91-0.98)	1.04 (0.95-1.14)	0.38
80+	0.93 (0.81-0.97)	1.0	
Among hematologic malignancy patients			
<30	0.59 (0.41-0.75)	0.67 (0.48-0.92)	0.01
30-39	0.46 (0.29-0.65)	0.53 (0.35-0.80)	0.003
40-49	0.36 (0.22-0.53)	0.42 (0.27-0.64)	<.001
50-59	0.54 (0.45-0.63)	0.64 (0.54-0.76)	<.001
60-69	0.69 (0.60-0.77)	0.83 (0.73-0.94)	0.004
70-69	0.77 (0.65-0.85)	0.89 (0.78-1.01)	0.07
80+	1.00 (0.82-1.00)	1.0	
**Sex**			
Female	0.73 (0.69-0.77)	1.0	
Male	0.76 (0.73-0.80)	1.03 (0.97-1.11)	0.33
**Primary Disease**			
Among 0-29 yr olds			
Solid tumor	0.73 (0.43-0.90)	1.0	
Hematologic malignancy	0.59 (0.41-0.75)	0.89 (0.55-1.44)	0.64
Among 30-39 yr olds			
Solid tumor	0.51 (0.36-0.66)	1.0	
Hematologic malignancy	0.46 (0.29-0.65)	1.01 (0.60-1.69)	0.98
Among 40-49 yr olds			
Solid tumor	0.73 (0.61-0.82)	1.0	
Hematologic malignancy	0.36 (0.22-0.53)	0.56 (0.36-0.88)	0.01
Among 50-59 yr olds			
Solid tumor	0.70 (0.61-0.78)	1.0	
Hematologic malignancy	0.54 (0.45-0.63)	0.89 (0.73-1.10)	0.28
Among 60-69 yr olds			
Solid tumor	0.92 (0.87-0.95)	1.0	
Hematologic malignancy	0.69 (0.60-0.77)	0.89 (0.78-1.01)	0.07
Among 70-79 yr olds			
Solid tumor	0.96 (0.91-0.98)	1.0	
Hematologic malignancy	0.77 (0.65-0.85)	0.91 (0.80-1.04)	0.15
Among 80+ yr olds			
Solid tumor	0.93 (0.81-0.97)	1.0	
Hematologic malignancy	1.00 (0.82-1.00)	1.07 (0.97-1.17)	0.18
**Last HCT**			
None	0.80 (0.77-0.83)	1.0	
<=1 year	0.61 (0.49-0.71)	0.93 (0.77-1.12)	0.46
>1 year	0.30 (0.21-0.42)	0.48 (0.34-0.69)	<.001
**Chemotherapy in past 30 days**			
No	0.76 (0.73-0.79)	1.0	
Yes	0.72 (0.67-0.77)	1.00 (0.93-1.08)	0.99
**Last IVIG**			
None	0.76 (0.73-0.79)	1.0	
<=16 wks	0.88 (0.53-0.98)	1.28 (0.90-1.83)	0.16
>16 wks	0.27 (0.14-0.44)	0.57 (0.33-0.99)	0.05

eFigure 1. Measles Seroprevalence Estimates and Adjusted Prevalence Ratio (PR) Estimates by Subgroup, for Sensitivity Analysis Including an Interaction Between Age Group and Disease Type. Squares represent measles seroprevalence estimates and brackets show the 95% CI for these estimates. The vertical dashed line shows the middle value (0.94) for the recommended range required for herd immunity (0.93-0.95). Prevalence ratio estimates from a multivariable model adjusting for age, sex, primary disease, HCT history, chemotherapy in the 30 days prior to sample collection, receipt of IVIG, and the interaction between age and primary disease are also shown. Abbreviations: HCT: hematopoietic cell transplant; IVIG: intravenous immunoglobulin treatment.

© 2021 Marquis SR et al. *JAMA Network Open.*

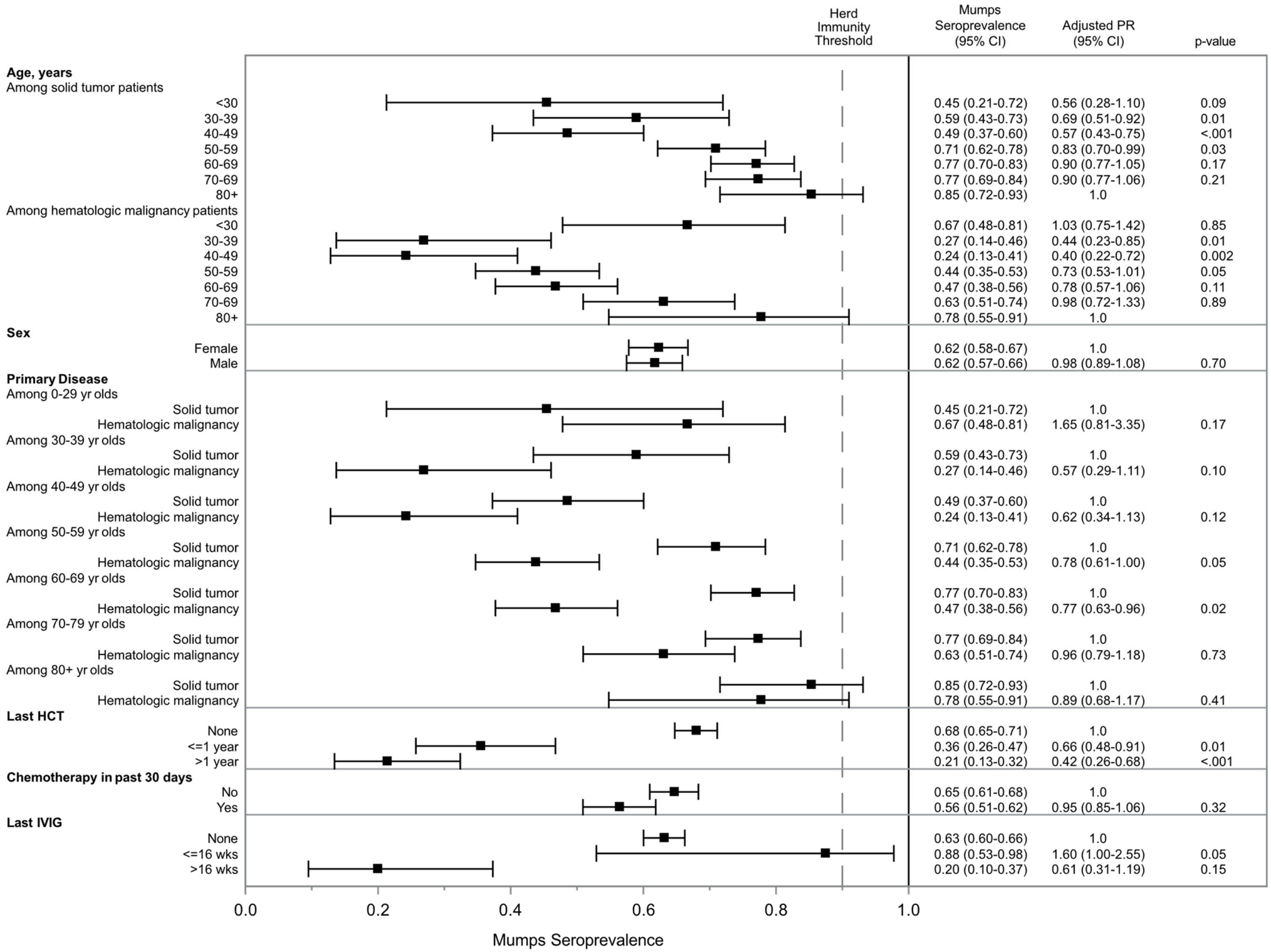

eFigure 2. Mumps Seroprevalence Estimates and Adjusted Prevalence Ratio (PR) Estimates by Subgroup, for Sensitivity Analysis Including an Interaction Between Age Group and Disease Type. Squares represent measles seroprevalence estimates and brackets show the 95% CI for these estimates. The vertical dashed line shows the middle value (0.90) for the recommended range required for herd immunity (0.88-0.92). Prevalence ratio estimates from a multivariable model adjusting for age, sex, primary disease, HCT history, chemotherapy in the 30 days prior to sample collection, receipt of IVIG, and the interaction between age and primary disease are also shown. Abbreviations: HCT: hematopoietic cell transplant; IVIG: intravenous immunoglobulin treatment.

© 2021 Marquis SR et al. *JAMA Network Open.*
